# 12-year survival analysis of 322 Hintegra total ankle arthroplasties from an independent center

**DOI:** 10.1080/17453674.2020.1751499

**Published:** 2020-04-14

**Authors:** Mina Jane Zafar, Thomas Kallemose, Mostafa Benyahia, Lars Bo Ebskov, Jeannette Østergaard Penny

**Affiliations:** a Department of Orthopedic Surgery, Hvidovre University Hospital, Denmark;; b Department of Orthopedics, Sahlgrenska University Hospital, Gothenburg, Sweden;; c Clinical Orthopedic Research Centre, Hvidovre Hospital, Copenhagen University Hospital, Denmark;; d Department of Orthopedic Surgery. University Hospital Zealand, Koege, Denmark

## Abstract

Background and purpose — Total ankle arthroplasties (TAAs) have larger revision rates than hip and knee implants. We examined the survival rates of our primary TAAs, and what different factors, including the cause of arthritis, affect the success and/or revision rate.

Patients and methods — From 2004 to 2016, 322 primary Hintegra TAAs were implanted: the 2nd generation implant from 2004 until mid-2007 and the 3rd generation from late 2007 to 2016. A Cox proportional hazards model evaluated sex, age, primary diagnosis, and implant generation, pre- and postoperative angles and implant position as risk factors for revision.

Results — 60 implants (19%) were revised, the majority (n = 34) due to loosening. The 5-year survival rate (95% CI) was 75% (69–82) and the 10-year survival rate was 68% (60–77). There was a reduced risk of revision, per degree of increased postoperative medial distal tibial angle at 0.84 (0.72–0.98) and preoperative talus angle at 0.95 (0.90–1.00), indicating that varus ankles may have a larger revision rate. Generation of implant, sex, primary diagnosis, and most pre- and postoperative radiological angles did not statistically affect revision risk.

Interpretation — Our revision rates are slightly above registry rates and well above those of the developer. Most were revised due to loosening; no difference was demonstrated with the 2 generations of implant used. Learning curve and a low threshold for revision could explain the high revision rate.

Arthritis in the ankle often develops earlier than in the hip or knee, and 70% have a traumatic etiology (Saltzman et al. 2005, Brown et al. 2006). Total ankle arthroplasty (TAA) can be indicated for severe arthritis in the ankle joint, but the anatomical preconditions, like a small surface area and high stress from compression and torque (Bouguecha et al. 2011, Kakkar and Siddique 2011), makes it less durable than hip and knee prosthetics. The Hintegra TAA, a 3-component mobile bearing, uncemented implant (Hintermann et al. 2004) is widely used and results from the development center demonstrate survival rates of 94% and 84% after 5 and 10 years’ follow-up (Barg et al. 2013). This is considerably more than the survival rates from national registries. Labek et al. (2011) demonstrated that development centers report only half of the revision rate that can be found in the few existing national registers. In a systematic review of primary Agility total ankle arthroplasty (DePuy Synthes Orthopedics, Warsaw, IN, USA), the author (Roukis 2012) found that the incidence of complications increased from 7% to 12%, in studies where the inventor was excluded. Similar results were found by Prissel and Roukis (2013), who found an increased incidence of complications from 6% to 13% in studies where the inventor or faculty consultants were excluded. These studies indicated the risk of selection (inventor) and publication (conflict of interest) bias.

Planning and surgical technique, including significant experience, are mandatory for a successful outcome. The better result from development centers may reflect, besides the above-mentioned bias, that there is a long learning curve and that the indication for revision surgery varies.

We examined the survival rates of primary Hintegra TAAs performed at Hvidovre Hospital, with revision rate as outcome. We report primary diagnosis for primary TAA and examine whether sex, generation of the implant, preoperative angles and implant position affect the revision rate.

## Patients and methods

The study is retrospective, and all operations were carried out at a specialized foot and ankle department, where the 2nd generation Hintegra TAA has been implanted since 2004 in low-demand elderly patients with severe arthritis.

The Hintegra TAA (Integra Life Sciences, Newdeal SA, Lyon, France) is an unconstrained, 3-component system that provides inversion–eversion stability. The Hintegra TAA includes 2 metallic components and an ultra-high-density polyethylene mobile bearing. The non-articulating surfaces have a porous coating with 20% porosity and are covered by cobalt-chromium and double hydroxyapatite coating (2nd generation Hintegra) or titanium fluid and hydroxyapatite (3rd generation Hintegra). Our institution changed from 2nd to 3rd generation mid-2007.

The patients were operated using a standard anterior approach, in accordance with the manufacturer’s instructions with relevant cutting guides and sizing regulations. The goal was neutral alignment in the AP view without collision laterally and the peak of the talus in side-view level between 40% and 50% from the frontal tibial edge. The operations were carried out using a tourniquet, and 1.5 g of cefuroxime was administered preoperatively. The foot was immobilized in a circular cast from the 2nd postoperative day for 3 weeks. The cast was converted to a removable boot (Don Joy type) for another 3 weeks, with weight-bearing as tolerated, followed by home physiotherapy if needed. Dalteparin 5000 I.E. s.c. was administered postoperatively for 3 to 5 days (this was prolonged in patients with risk factors).

### Data collection

The in-house operation booking system (Orbit, Evry Healthcare Systems AB, Kristianstad, Sweden) and the hospital’s local registration system were searched for primary and secondary insertion of Hintegra TAA, using the designated codes for ankle implants between 2004 and 2016. This search yielded 322 surgeries from 2004 to 2016 using primary Hintegra TAA.

All had a patient record review, and an assessment of the radiographs. Revisions were noted, as were the reasons for revision and the revision type. The revisions were defined according to Henricson et al. (2011a): “A revision of the TAA is defined as removal or exchange of one or more of the prosthetic components with the exception of incidental exchange of the polyethylene insert.” For this study, only the 1st revision was included. We did not record minor wound complications etc. that did not lead to additional surgery. Aseptic loosening was defined as the failure of the bond, between an implant and bone, in the absence of infection, defined on radiographic findings of radiolucent lines around the implant and/or peroperatively, as lack of bony ingrowth.

Primary diagnosis (post-traumatic, primary osteoarthritis [OA], rheumatoid arthritis [RA], and other), sex, age at surgery, time since the surgery, and the generation of the prosthesis were registered.

### Radiology

**Figure 1.**
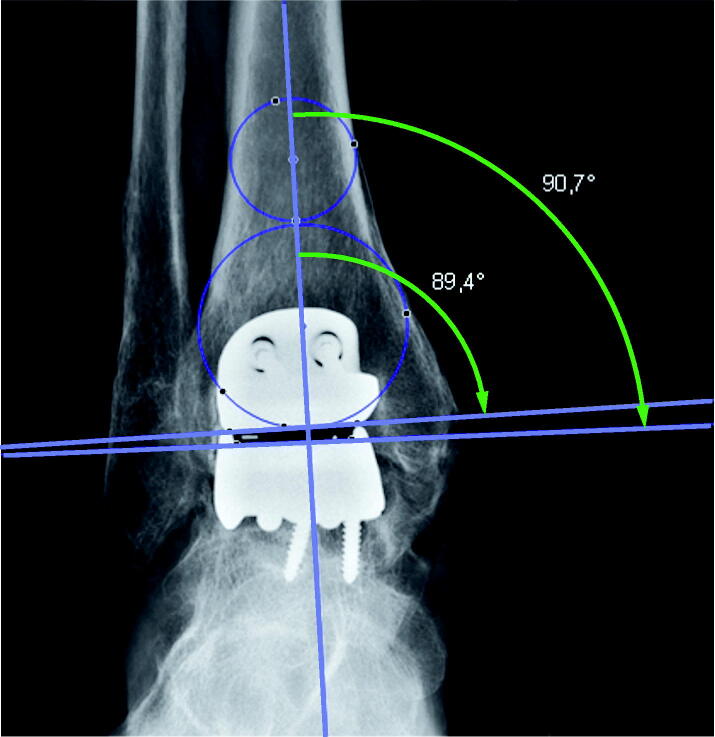
The center of the tibial plateau was determined by drawing a circle within the medial and lateral cortex. A second circle fit inside the distal tibia between the medial and lateral cortex and touched the plafond distally. The mechanical axis goes through both the center of the distal tibia and the center of the talus. A line marking the tibial plateau/distal tibial component intersected the mechanical axis for the medial distal tibial (MDTA; small arch) angle. The medial talus (large arch) angle was measured from a transecting line, tracing the superior talus/talar component. An angle above 90° is a valgus angle and below 90° is a varus angle.

The preoperative radiographs were digital and varying in length. For the preoperative images, images within 6 months prior to the surgery were accepted. We attempted to use the most recent and aimed to use a mortise view (mortise view was missing in a few cases where a straight AP was used). The postoperative radiographs were not strictly standardized but contained a mixture of weight-bearing and non-weight-bearing images. The postoperative implant position was accepted in images up to 4 months postoperatively. The valgus/varus position pre- and postoperatively was measured on short images using medial distal tibial angle (MDTA) and medial talus angles (Figure 1) according to Barg et al. (2012).

**Figure 2.**
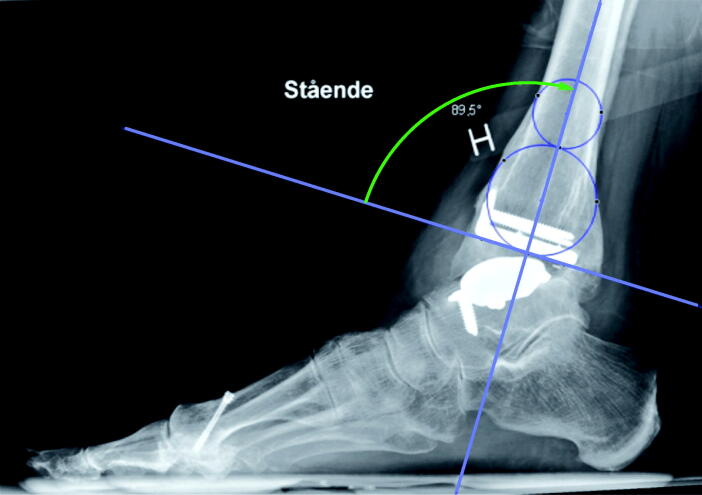
Markings to calculate the anterior distal tibial angle tibial plateau/distal tibial component (ADTA; green arch). For tibial axis, see Figure 1.

The horizontal line of the anterior distal tibial angle (ADTA; Figure 2) was defined by the most distal anterior and posterior bony points preoperatively, and by the distal tibial component postoperatively.

### Statistics

Survival rates by Kaplan–Meier curves were stratified by the generation of the implant. The analysis of risk factors for revision rate used the Cox proportional hazards model, with revision as outcome. The variables of interest were: time since surgery, age at surgery, sex, placement of the TAA (MDTA and ADTA as well as medial talus angle), preoperative alignment of the ankle, generation of the TAA, and primary diagnosis (post-traumatic, primary OA, RA, and other). The model included a combination of the generation of the TAA and the time the specific generation had been in use, thereby allowing the effect of time within each prosthesis type.

Hazard ratios and 95% confidence intervals (CI) were determined. P-values below 0.05 were considered statistically significant. The analysis was carried out in R 3.0.2 (R Foundation for Statistical Computing, Vienna, Austria).

### Ethics, funding, data sharing, and potential conflicts of interest

As the study was based on registry data, ethical approval is not needed according to Danish law. This research did not receive grants from any funding agency. The data are available from the corresponding author. We have no conflict of interest to declare.

## Results

**Figure 3.**
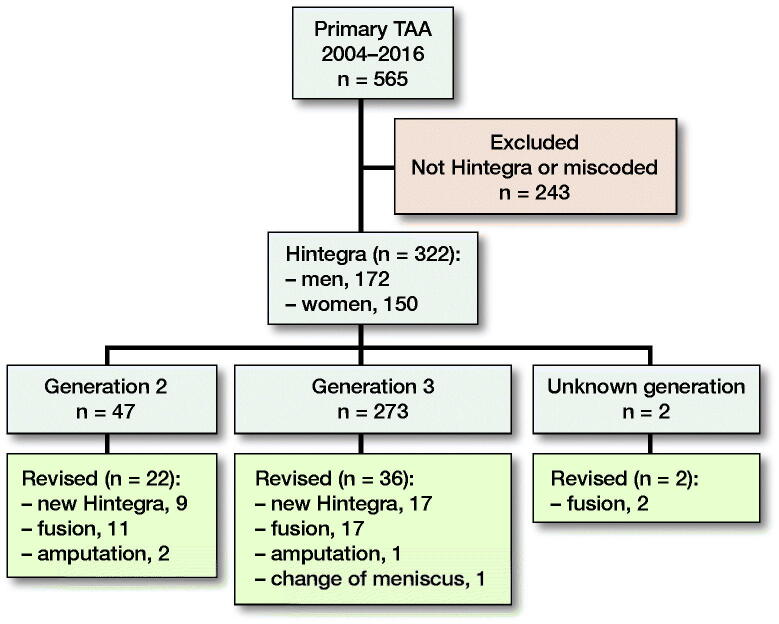
Flow chart.

The search identified 322 Hintegra TAA (Figure 3). 47 were 2nd generation and 273 were 3rd generation. 2 were unknown generation but they were implanted around the time we changed from 2nd to 3rd generation. 150 patients were females and 172 males. 13 were bilateral. Mean age was 60 years (24–81).

60% of the 322 primary implants had posttraumatic OA, 31% primary OA, 9% RA, and < 1% other, which had no statistically significant impact on revision risk.

60 implants were revised. The reasons for revision were 34 cases with suspected aseptic loosening, aseptic loosening with or without cyst formation as primary indication (8 of these cases with radiographic cyst formation/mechanical loosening), 7 cases of infection, 6 cases of malalignment, 3 cases of persistent pain, 3 cases of fracture, and 7 cases of other reasons.

22 of 47 2nd-generation Hintegra were revised: 9 to new Hintegra (5 partly and 4 fully revised), 11 were revised to fusions, and 2 were amputated due to problems with wound healing and chronic nerve pain. 36 of 274 3rd-generation were revised to 17 new Hintegra (2 fully revised, 15 partly revised), 17 to fusion, 1 was amputated for unknown reasons, and 1 had a change of a fractured meniscus. 2 unknown generation implants were both revised to fusion.

Additional surgery after the primary Hintegra implant was recorded in 40 cases but was not registered as revision as the implants were left intact. The procedures included cheilectomy/osteophyte removal (n = 4), decompression medial or lateral (n = 8), bone grafting of cyst (n = 3), ligament reconstruction (n = 2), fracture repair (n = 2), osteotomy (n = 6), wound infection (n = 5), a mixture of these above (n = 6), and other (n = 9).

The operations were performed by 8 different surgeons and 13 were operated by unknown surgeons (Table 1).

**Table 1.** 322 total ankle arthroplasties (TTAs) distributed among 8 different surgeons and subsequent revision surgeries, 13 unknowns

Surgeon	1	2	3	4	5	6	7	8	Unknown
Years active	2008–2014	2005–2010	2014–2016	2006–2016	2010–2016	2004–2011	2007–2016	2012	2003–2007
Primary TAAs	21	11	14	117	67	38	40	1	13
Revision, n	1	4	0	11	11	23	3	1	6

**Figure 4.**
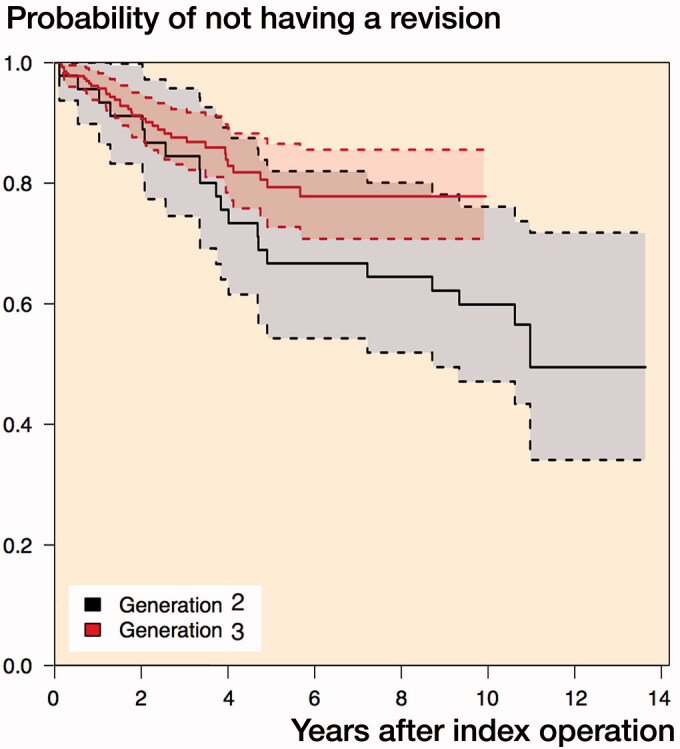
Kaplan–Meier plot of the survival rates of generation 2 and generation 3.

The survival at 5 and 10 years was 0.75 (CI 0.69–0.82) and 0.68 (CI 0.60–0.77) respectively. For the 2nd generation, the 5- and 10-year survival was 0.67 (CI 0.54–0.82) and 0.60 (CI 0.47–0.76) and for the 3rd generation the 5- and 10-year survival was 0.79 (CI 0.73–0.87) and 0.78 (CI 0.71–0.86) respectively (Figure 4).

In the Kaplan–Meier plot, the survival rate of generation 2 was slightly below that of generation 3. The univariate analysis found a non-statistically significant, lower expected risk of revision of the 3rd generation than the 2nd of 0.60 (0.33–1.08). The multivariate analysis found a similar, non-statistically significant effect size of 0.56 (0.23–1.3).

The multivariate Cox proportional hazards model (Table 2) found a reduced risk of revision for each increased degree of the postoperatively MDTA, 0.84 (0.72–0.99), suggesting that a valgus position of the implant is safer than a varus position. Similarly, the small effect of reduced risk of revision of 0.95 (0.90–1.00) per degree of increased preoperative medial talus angle suggests that patients with pre-existing varus before TAA surgery had revision more frequently than valgus ankles.

**Table 2.** The Cox proportional hazard multivariate analysis of risk for revision

Factor	Hazard ratio (95% CI)
Male sex	1.10 (0.50–2.4)
Age	0.98 (0.95–1.0)
Generation 3	0.56 (0.23–1.3)
Post-trauma	1.23 (0.48–3.2)
Post-infection	1.26 (0.10–22)
Rheumatoid arthritis	1.26 (0.31–5.1)
Angle pre-Hintegra (per 1°)	
MDTA	1.05 (0.95–1.2)
Medial talus angle	0.95 (0.90–1.0)
ADTA	1.01 (0.94–1.1)
Angle post-Hintegra (per 1°)	
MDTA	0.84 (0.72–0.98)
Medial talus angle	1.02 (0.90–1.2)
ADTA	1.04 (0.93–1.2)

The Cox proportional hazard multivariate analysis shows changes in risk for revision when the variable is increased by 1 unit. When the MDTA and medial talus variable are increased by 1° it means that the ankle goes towards a valgus position. Whether it results in an increase or reduction of the risk depends on the hazard ratio size; if this is less than 1, there is a reduced risk and if it is more than 1, it is an increased risk. MDTA = medial distal tibial angle, ADTA = anterior distal tibial angle.

No risk was found for sex, age, the primary diagnosis, and the remaining pre- and postoperative angles in the multivariate analysis but increasing age at surgery was associated with a lower risk of revision in the univariate analyses, 0.97 (CI 0.94–0.99).

The results were similar by restricting them to revisions due to osteolysis/aseptic loosening and malalignment alone.

## Discussion

The primary aim of our study was to analyze the survival rates of primary Hintegra TAA. With a total survival rate of 75% after 5 years and 60% after 10 years, our results are inferior compared with the results reported by Barg et al. (2013) where overall survival rates of 634 patients were 94% and 84% after 5 and 10 years, respectively. The Swedish Ankle Registry annual report for 2014 reports a 1.9 increased risk of revision compared with the development center, but the numbers in the register are very low and the difference does not reach statistical significance. Willegger et al. (2013) found that 10 of 16 Hintegra TAA implants had survived after 4 years and suggests that publications by implant inventors show a tendency towards superior results. Our 3rd-generation Hintegra univariate risk of revision was 0.60 (0.33–1.08), compared with 2nd-generation Hintegra. The difference is not statistically significant but could reflect a type II error since the effect size (0.60 vs. 0.56) remained relatively unchanged. However, as the majority of the surgeons had their learning curve before the introduction of the 3rd-generation Hintegra the effect size could likely reflect learning curve bias rather than type II error of the design difference. This is supported by Roukis et al. (2016), who pointed out that design may not be so important as previously believed, as much as the weighted mean survival after the 1st-generation TAA prostheses was 0.76 at 10 years, for 2nd generation 0.83 and for 3rd generation 0.83.

In our study there was a small number of operations per surgeon (6 of 8 surgeons had 40 TAAs or below) compared with the developer center. It is well established that TAA surgery involves a significant learning curve (Haskell and Mann 2004). This may explain the higher revision rate found in this study. Yang et al. (2019) reported, in a large series but single surgeon study, good survival rates of 92% at a mean 6.4 years.

The 5-year survival rate of TAAs in registries is between 93% and 78% (Bartel 2015). The Australian Registry (2019) with 442 primary Hintegra implants reports a 5-year cumulative revision rate of 11%, in line with the other implants in the register. The 5-year cumulative revision rate of all TAAs in the UK is 7%, but the British Orthopaedic Association itself suspects underreporting of revisions to the registry (UK National Joint Registry 2019).

The Hintegra implants in the New Zealand register are limited and with very wide 95% confidence intervals for revision. Both the NZ and the Australian Registry use revision per component year. In registries with many “young” implants this will reflect the early revisions due to infection etc. but may not capture many of the aseptic loosenings that occur later and may give a different profile than, e.g., the Swedish registry (NZJR 2013).

The revision rates in our study are not only considerably higher than those of the developer but also above those of the registries. Our survival rates are lower than the weighted mean survival after 1st-generation TAA. The registries, however, are also flawed. The UK suspects a completion rate below 90% but has no data to validate this yet. The Finnish rate is just above 90% (Skyttä and Koivu 2010), and most others are not disclosed. The French registry registers below 80% (Besse et al. 2010) and misses many from small-volume centers, and the risk of underreporting of revision is high in the registry data.

The strength of our study is the unedited volume of a single center with multiple surgeons.

In our study, the indications for revision in more than half of cases were suspected aseptic loosening because of diffuse loosening or cyst formation. Approximately half were revised to a new TAA and half to a fusion. The prosthetic design may not be the most important factor influencing long-term survival. Proper patient selection and optimal prosthetic implantation including a perfect balanced ankle are probably more important, as regards the impact on the survival rate, than the indication for revision.

The high revision rate may reflect that our center tends to have an aggressive revision approach, if we suspect aseptic loosening and/or cyst formation. We believe it facilitates revision surgery without the need for more substantial bone grafting and often prevents fusion. As a referral center, we treat patients from other centers, where large cyst formation and possible loosening are typically accepted if the patient does not have significant pain. It is our experience that this pending strategy often leads to complex fusions with large structural allografts. The problem, when comparing survival and revision rates, is that the criteria for revision are not formalized, nor are the different ways of reporting the function of the patients.

We found patients with high talar angle preoperatively and high MDTA postoperatively to have fewer revisions. This is in line with the findings of Henricson and Ågren (2007) and further discussed by Coetzee (2008). However, Lee et al. (2018) showed in their comparison of the survival rate on their 144 Hintegra patients that the long-term outcome was equally as good regardless of the preoperative alignment (up to 20°) as long as the postoperative alignment was neutral. Considering the non-standardized and retrospective radiographic set-up available in our study these results should be evaluated with care; nonetheless, optimal alignment of the prosthesis should logically reduce the possible factors that may lead to aseptic loosening and pain.

The univariate analysis suggested a reduction of revision risk with increasing age, in line with the findings of Henricson et al. (2011b), but the effect was not statistically significant in the multivariate analysis. It does, however, mirror the results from the Australian (2019) and New Zealand Ankle Registry (NZJR 2018) and probably reflects the lower demands for function, as well as a disinclination to perform surgery on the elderly with their associated somatic risks (NZJR 2013). However, a short-term study only found slightly lower and non-significant functional levels and slightly more comorbidities for the elderly (Demetracopoulos et al. 2015). Johnson-Lynn et al. (2018) did not show any definite association between age and revision, and discussed that this was likely due to the small total number of revisions at their 5-year follow-up of 106 patients.

The strength of our study is the unedited volume of a single center. It is the largest independent Hintegra patient group, with a 10-year survival analysis reported.

The drawback of the study is the retrospective design. The assumed primary diagnosis was not always present in the patient record and the exact cause of revision is, in some cases, not precisely described. Cysts are known to affect the revision rate (Labek et al. 2011). It was not our impression that cyst formation was prominent but, due to a new IT provider, the old images are not available for confirmation of this. We are also limited retrospectively in having only plain radiographs and not CT images. Hanna et al. (2007) showed that radiographs are not sufficient to diagnose cyst formation or osteolysis. Deleu et al. (2014) indicated 24 cases of osteolysis in 50 patients with the Hintegra prosthesis. This number, larger than ours, can most likely be explained by our limitation in radiographic imaging. We do agree with Yang et al. (2019), who had 31 cases of peri-prosthetic osteolysis in their study of 242 TAA, that the prevention of peri-prosthetic osteolysis should be a priority for TAA.

Another drawback is that the postoperative radiographs were not standardized. Only a few radiographs were AP rather than mortise view. We have been unable to find literature concerning the effect of axial rotation on the distal tibial angle but, with only these few exceptions from standard view, we find that our results are valid and further supported by being in agreement with previous findings (Henricson and Ågren 2007, Coetzee 2008).

In conclusion, we found revision rates slightly above the registries, but well above those of the development center. More than half were revised due to suspected loosening with aseptic loosening or cysts. No difference was demonstrated between the 2 generations of implant used. The high number of surgeons with a low number of operations (learning-curve surgeons) and an aggressive approach to treating aseptic loosening and suspected aseptic loosening might explain the inferior survival rate in this study.
